# Integrated plasmonic circuitry on a vertical-cavity surface-emitting semiconductor laser platform

**DOI:** 10.1038/ncomms12409

**Published:** 2016-08-05

**Authors:** Cillian P. T. McPolin, Jean-Sebastien Bouillard, Sebastien Vilain, Alexey V. Krasavin, Wayne Dickson, Daniel O'Connor, Gregory A. Wurtz, John Justice, Brian Corbett, Anatoly V. Zayats

**Affiliations:** 1 Department of Physics, King's College London, Strand, London WC2R 2LS, UK; 2 Tyndall National Institute, Lee Maltings, Cork T12R5CP, Ireland

## Abstract

Integrated plasmonic sources and detectors are imperative in the practical development of plasmonic circuitry for bio- and chemical sensing, nanoscale optical information processing, as well as transducers for high-density optical data storage. Here we show that vertical-cavity surface-emitting lasers (VCSELs) can be employed as an on-chip, electrically pumped source or detector of plasmonic signals, when operated in forward or reverse bias, respectively. To this end, we experimentally demonstrate surface plasmon polariton excitation, waveguiding, frequency conversion and detection on a VCSEL-based plasmonic platform. The coupling efficiency of the VCSEL emission to waveguided surface plasmon polariton modes has been optimized using asymmetric plasmonic nanostructures. The plasmonic VCSEL platform validated here is a viable solution for practical realizations of plasmonic functionalities for various applications, such as those requiring sub-wavelength field confinement, refractive index sensitivity or optical near-field transduction with electrically driven sources, thus enabling the realization of on-chip optical communication and lab-on-a-chip devices.

## Abstract (web summary)

Integrated plasmonic sources are crucial in the development of plasmonic circuitry. Here, McPolin *et al*. show that vertical-cavity surface-emitting lasers can be employed as an on-chip, electrically pumped source or detector of plasmonic signals and also demonstrate waveguiding and frequency conversion on this platform.

## Graphical abstract

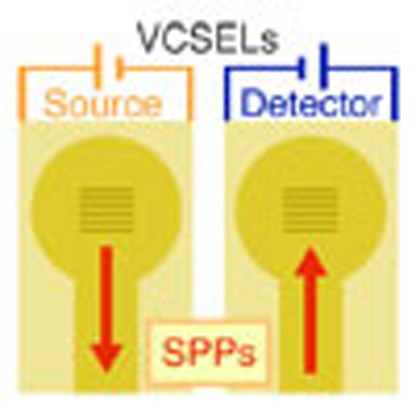

Surface plasmon polaritons (SPPs) at metal-dielectric interfaces offer a means of constructing compact nanophotonic components due to their confined electromagnetic fields^1^. Consequently, plasmonic technology has a wide range of potential applications, in areas as diverse as optical data processing and storage, information transmission and bio-, chemical and environmental sensing. In order to develop functional plasmonic circuitry, a set of components is required for the active control of signals, including SPP sources, detectors, switches and modulators that, when combined with passive waveguides, allow for the full manipulation of optical signals on the nanoscale^2^. Due to their compatibility with Si and other dielectric photonic circuitry, passive plasmonic components, together with various switches and modulators, have recently experienced rapid progress. However, despite their fundamental importance in plasmonic circuitry, electrically driven plasmonic sources and detectors have not been developed to the same extent, which prevents the realization of an integrated, on-chip plasmonic platform.

In this paper, we demonstrate a vertical-cavity surface-emitting laser (VCSEL) based platform for the on-chip generation, manipulation and detection of plasmonic signals. VCSELs hold considerable promise in this regard, as they are compact, inexpensive, power-efficient and reliable^3^. In addition, they have previously been used to illustrate SPP excitation^4^,^5^,^6^. However, in order to successfully establish a robust platform for a wide range of applications, a variety of on-chip components are required that permit plasmonic signals to be effectively controlled. In this context, we demonstrate an on-a-VCSEL-chip integrated SPP platform. The proposed circuitry utilizes laser diodes that are based on 850 nm wavelength VCSELs directly coupled to a plasmonic platform that incorporates excitation and waveguiding structures, including directional couplers, SPP splitters and Mach–Zehnder interferometers. To further advance this integrated plasmonic platform, we also demonstrate SPP frequency conversion in dielectric loaded waveguides and VCSEL-based plasmonic signal detection. The plasmonic circuitry developed here facilitates high-integration of nanophotonic applications, thus presenting immediate opportunities for sensing, data storage and signal transmission.

## Results

### Plasmonic VCSEL characteristics

**Figure 1:**
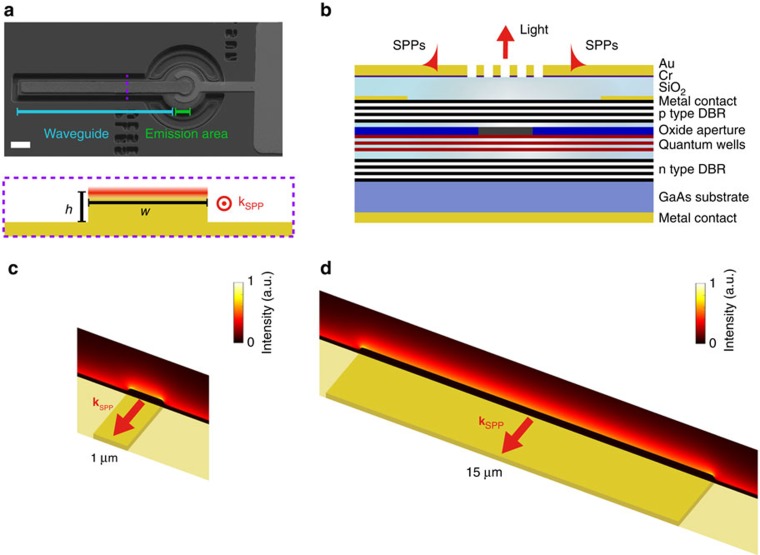
Plasmonic VCSEL. (**a**) Scanning electron microscope image of a plasmonic VCSEL and a schematic of a cross-section across the stripe waveguide with width *w* and height *h*. **k**_SPP_ is the SPP wavevector, directed parallel to the long axis of the waveguide. Scale bar, 20 μm. (**b**) Cross-section of a plasmonic VCSEL based on a diode emitting at a wavelength of 850 nm, with an additional gold layer that supports SPP modes. The emission area, and hence SPP excitation region, is defined by the underlying position of the oxide aperture. (**c**,**d**) The SPP mode intensity distributions overlaid on the waveguide schematics, obtained from the eigenmode simulations for the Au stripe waveguides with (**c**) *w*=1 μm and (**d**) *w*=10 μm, where *h*=100 nm in both cases. a.u., arbitrary units.

The plasmonic VCSELs (Fig. 1a,b) were fabricated using a standard process (see Methods for details), and exhibited threshold currents of ∼1 mA, similar to the reference VCSELs (without plasmonic layers). The reference VCSELs' power was on the order of milliwatts, with emission in a fundamental transverse mode that possessed an experimentally verified Gaussian profile, thus confirming the suitability of these lasers for direct SPP excitation via one-dimensional nanoslit gratings, which are widely employed for SPP excitation with free-space light. The total power emitted into the far field from the plasmonic VCSELs is ∼1/200 of the power emitted from the reference VCSELs, due to the presence of the metallic layers (Supplementary Fig. 1).

In order to demonstrate plasmonic functionality, the VCSELs were integrated with an additional Au layer (200 nm thickness) to provide an SPP supporting surface into which micrometres-wide, multimode plasmonic stripe waveguides were patterned. A 20 nm thin layer of chromium was used as both an adhesion layer and to suppress SPP modes on the lower interface of the Au film. Moreover, the relatively large thickness of the Au layer, together with the Cr film, prevents the leakage of SPP modes at the Au–air interface back into the VCSEL cavity.

To enable on-chip SPP excitation, nanoslit gratings were ion milled through the top metal layers, directly above the VCSEL oxide aperture (Fig. 1b), with the long axis orthogonal to the direction of the plasmonic waveguide. Upon applying a forward bias, the resulting optical emission is phase matched to SPPs via scattering from the gratings, thereby launching SPP modes that propagate at the Au–air interface. The SPP intensity depends on the coupling efficiency of the grating and increases linearly with the intensity of the VCSEL emission. In addition, the maximum intensity emitted by each VCSEL remains well below the damage threshold for the plasmonic layers.

The measured far-field spectra of both reference and plasmonic VCSELs display a dominant single emission line (wavelength 845 nm) associated with single transverse and longitudinal mode behaviour at drive currents of up to 3 mA (Supplementary Fig. 2). A secondary emission line appears at currents >4 mA, with a 20 dB difference in power compared with the primary emission wavelength. This behaviour indicates that the devices possess a single spatial mode profile at low currents with a second spatial mode emerging at higher currents. As the active region is protected, laser instability was not observed, and the spectral properties of both the plasmonic and reference VCSELs remain the same at similar threshold currents. Thus, the spectral and modal characteristics of the VCSELs, together with the threshold currents, are unaffected by the integration of plasmonic layers with gratings.

### SPP excitation and waveguiding

The integrated SPP waveguides were numerically simulated to determine the profiles of guided plasmonic modes. Figure 1c,d shows the SPP power flow for eigenmode calculations of two different stripe widths, highlighting the fact that most of the mode energy resides on the flat interface. A large stripe height, *h*, also ensures the interaction between the waveguided modes and the lower Au surface is minimized. While some field localization is observed at the corners, it does not have a significant role in determining the mode profile. Furthermore, rounding the edges of the stripes in the numerical simulation, to more closely resemble the fabricated structures, reduces this effect.

**Figure 2:**
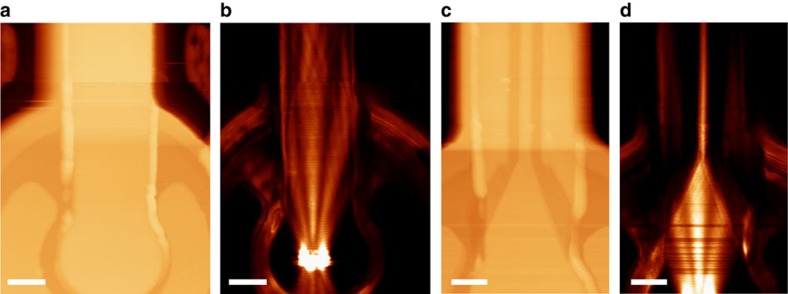
SPP waveguiding on plasmonic VCSELs. (**a**,**c**) Topography and (**b**,**d**), near-field intensity distributions for (**a**,**b**) multimode waveguide (*w*=10 μm, *h*=100 nm) and (**c**,**d**) single-mode waveguide (*w*=1 μm, *h*=100 nm). Each near-field image is composed of two scans of adjacent regions, allowing a large area to be mapped. Scale bar, 8 μm (**a**,**b**); Scale bar, 5.4 μm (**c**,**d**).

SPP excitation and waveguiding on the plasmonic VCSELs is experimentally demonstrated (Fig. 2) using scanning near-field optical microscopy^7^,^8^. The VCSEL emission incident on the gratings excites SPPs that are visible in the near-field images as high intensity beams that decay exponentially with distance from the emission area. These features are absent from the far-field images obtained when the scanning near-field optical microscope probe was retracted from the surface, hence confirming the localization of optical energy in the near field. The propagation of SPPs away from the emission area and onto the stripe is reasonably efficient as the stripe width, *w*, is large, yielding a complex intensity profile due to the reflection of SPPs at the boundaries of the metal film (Fig. 2b). Thus, the gold slab adjacent to the emission area forms a multimode stripe plasmonic waveguide, with the number of supported modes depending on its width. Mode formation in these waveguides may be considered in terms of SPP reflection at the stripe boundaries, together with edge effects^9^,^10^,^11^.

Further control over the SPP mode profile and propagation can be obtained by coupling the plasmonic signal to a single-mode waveguide. Using focused ion beam milling, 1 μm wide stripes were created on the metal layer, which only support a single mode at the SPP wavelength of ∼835 nm, corresponding to the 850 nm free-space wavelength of the VCSEL emission (Fig. 2c,d). As shown in the near-field image of Fig. 2d, the tapering of a 10 μm wide stripe resulted in the effective filtering of the plasmonic signal as it propagated onto the single-mode waveguide, after which the mode maintained a constant lateral profile. A small width also permits a high integration density for waveguiding components. The numerically calculated effective index 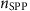 of the fundamental plasmonic modes supported by both 10 and 1 μm wide waveguides (where the SPP wavevector is directed parallel to the waveguide's long axis) approximately equals the index of SPPs on an infinite, smooth film, 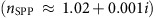 as determined for a free-space wavelength of 850 nm.

**Figure 3:**
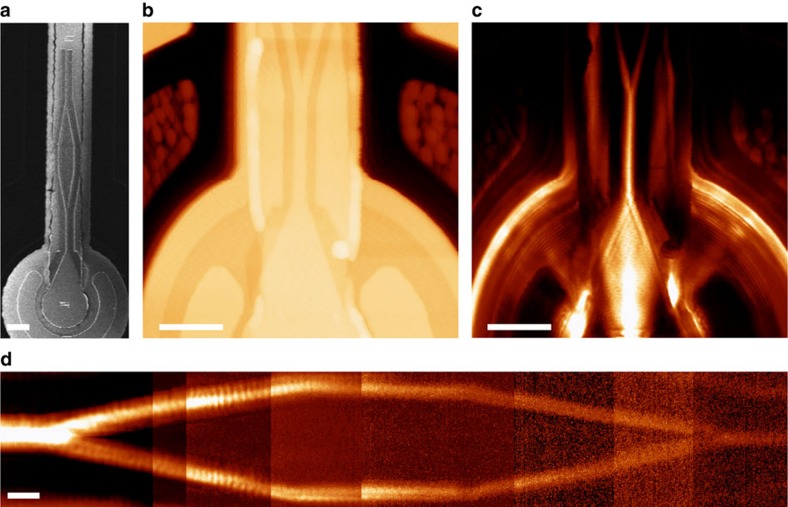
Mach–Zehnder interferometer integrated on a VCSEL. (**a**) Scanning electron microscope image of an MZI. (**b**,**c**) Topography and near-field intensity distribution of the coupling and splitting branches of the MZI. (**d**) Near-field intensity distribution in the MZI showing splitting and recombination of the SPP modes. The near-field optical microscope scanning range is smaller than the MZI length, therefore; several images were measured in a sequence and subsequently pasted together. Scale bar, 10 μm in (**a**); Scale bar, 11 μm in (**b**); Scale bar, 11 μm in (**c**); Scale bar, 1.6 μm in (**d**).

By employing single-mode waveguides, more complex structures may also be implemented on plasmonic VCSELs, such as a Mach–Zehnder interferometer (MZI) (Fig. 3). In this case, the plasmonic signal splits into the two separate waveguide branches that extend for a few micrometres before subsequently merging, allowing the SPP waves to recombine (Fig. 3d). The output SPP intensity depends upon the phase difference between the signals in the two MZI branches, which may be either actively modulated (for example thermally, electrically or all-optically) or passively, when specific analytes are adsorbed onto the exposed metal surface^2^. As a result, the MZI geometry can be used for both modulation and sensing in SPP circuitry.

**Figure 4:**
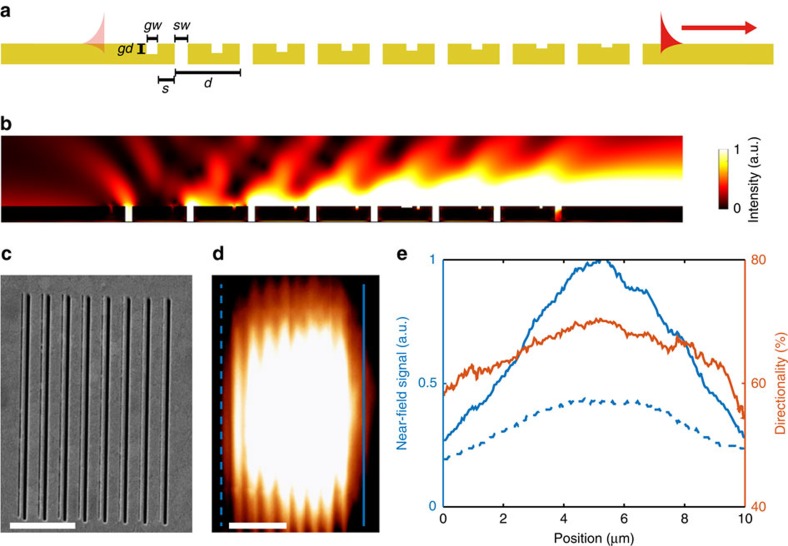
Directional SPP excitation on a VCSEL. (**a**) Schematic of a slit-groove coupler with period *d*, slit width *sw*, groove width *gw*, separation *s* and groove depth *gd*. (**b**) Simulated intensity distribution of the SPP modes excited under normal illumination from below for the structure with parameters *d*=800 nm, *sw*=100 nm, *gw*=50 nm and *s*=150 nm. A linear decrease of groove depth is introduced from left to right, decreasing the SPP reflectivity. The illuminating light is polarized perpendicularly to the grooves. (**c**) Scanning electron microscope image of the slit-groove coupler milled into a plasmonic VCSEL. (**d**) Near-field intensity distribution measured above the coupler and the SPP waveguide with the cross-sections shown in **e**. (**e**) The cross-sections of the SPP intensity profile to the right (solid blue line) and left (dashed blue line) from the coupler, measured from the scanning near-field optical microscopy image in **d**, and the directionality parameter (red line). Directionality is defined as the ratio of the SPP power flowing to the right divided by the total SPP power. Scale bars, 2.5 μm in (**c**,**d**). a.u., arbitrary units.

Due to the symmetric in-plane scattering cross-section of the nanoslits, normal incidence illumination from the VCSEL on the one-dimensional grating structures launches two symmetric waveguided SPP modes propagating in opposing directions, perpendicular to the slits in the grating (Fig. 2b). Such bidirectional launching of SPPs is advantageous for particular applications, nonetheless more precise control over the direction of excitation is often required. In the case of a single waveguide VCSEL (Fig. 1a), SPPs that are not propagating towards the waveguide essentially constitute a loss channel. Hence, to excite a directional SPP signal, a slit-groove structure was employed (Fig. 4). Asymmetric SPP excitation occurs in this structure due to the reflection of SPPs by the grooves, which may be understood in terms of coupling to modes within the groove. Considering a single slit with an adjacent groove, SPPs launched by the slit may directly transmit across the groove, or be coupled down into the groove. These slot modes subsequently re-scatter at the groove exit, also launching single interface SPPs in the process. As a result of the phase difference acquired through reflections and propagation, SPPs excited via the groove may destructively interfere with SPPs directly transmitted across the groove, allowing the structure to function as a mirror for selected SPP frequencies^12^. Furthermore, periodically repeating this unit cell increases the total power coupled to SPP modes. In effect, a groove SPP-reflection grating was integrated with a slit SPP-excitation grating, ensuring that the coupler dimensions are minimized. Furthermore, a gradual decrease in groove depth across the structure effectively decreases the SPP reflectivity and reduces the undesired reflection and scattering losses experienced by modes already travelling in the desired direction. To determine the optimal geometrical parameters, a Monte Carlo optimization process was employed.

Using optimized geometrical parameters, numerical calculations show that such gratings allow for up to 90% of the total SPP power to propagate in a unique direction, towards the waveguide (Fig. 4b). Experimentally, this value reaches almost 70%, as measured from the near-field image (Fig. 4d,e); the difference between the experimentally measured and simulated directionalities may be partially attributed to a deviation from the designed geometrical parameters.

The laser coupling efficiency to the SPP mode may be estimated from the power exiting the slit apertures of the grating divided by the power in the SPP mode at the distance 8 μm to the right of the grating, taking into account SPP propagation loss. Using this definition, the grating described in Fig. 4a has a conversion efficiency of almost 50%, as determined from the simulations. A Cr adhesion layer prevents SPP excitation on the Au–oxide interface and, while reducing the optical transmission (∼25% reduction in transmission), does not impact upon the directionality or the conversion efficiency (this Cr layer is included in the simulation in Fig. 4b). Consequently, on-chip, asymmetric excitation of SPP modes has been successfully demonstrated. Alternatively, plasmonic crystal structures may also be implemented on the VCSEL platform to further control the excitation, direction and shape of SPP beams^13^,^14^.

### SPP frequency conversion

Frequency conversion provides a route to novel wavelength sources, in addition to being an important process for many applications related to all-optical networks and sensing^15^,^16^. It has recently been shown that the SPP-excited lasing in a plasmonic system has a much higher efficiency than in the case of free-space light excitation^17^. In this context, SPP frequency conversion has been achieved on the VCSEL platform via a dielectric-loaded SPP waveguide approach by including a layer of emitting quantum dots on top of the stripe SPP waveguide. Using spin-coating, a 200 nm thin film of PMMA, doped with 5 wt% concentration of PbS quantum dots, was deposited on the chip containing a plasmonic VCSEL. For this particular demonstration, the quantum dots were specifically chosen to emit at the telecom-relevant wavelength range around 1,500 nm. The SPP waveguide modes generated by the VCSEL emission efficiently excite the quantum dots that reside on the waveguide, which subsequently decay directly into plasmonic modes, ensuring the excitation of an SPP waveguided mode with a wavelength determined by the quantum dot emission spectrum.

**Figure 5:**
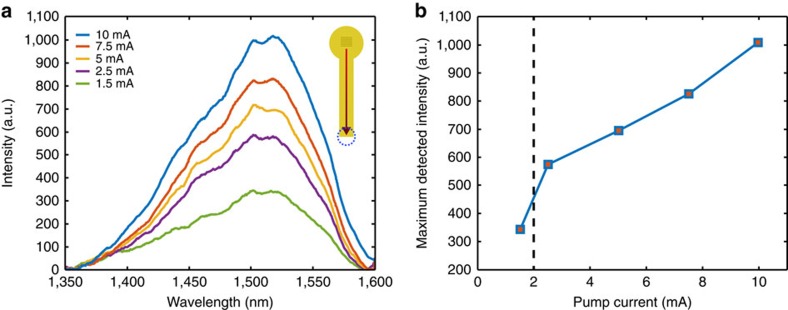
SPP frequency conversion on a VCSEL. (**a**) SPP scattering spectra for different VCSEL driving currents, measured from the dielectric-loaded SPP (DLSPP) waveguide doped with PbS quantum dots with the peak emission wavelength of ∼1,500 nm. The inset shows a schematic with the collection area marked by a blue circle, where SPPs out-couple due to scattering at the end of the DLSPP waveguide. (**b**) The dependence of the intensity at 1,515 nm on the VCSEL driving current. The threshold current for this VCSEL was about 2 mA, as shown by the dashed line. a.u., arbitrary units.

Frequency conversion was experimentally verified by collecting the light that originated from scattering of luminescence-driven SPPs at the end of a gold stripe, after the SPP signal had traversed the waveguide and interacted with the emitters. The observed broad spectrum is consistent with the expected quantum dot emission (Fig. 5a). Moreover, the maximum detected signal increases linearly with applied current, once the lasing threshold current has been exceeded, further confirming the SPP–SPP frequency conversion mechanism (Fig. 5b). Hence, the experiment illustrates on-chip manipulation of the SPP wavelength, a key process for plasmonic technology.

### SPP detection

SPP detectors are one of the basic components required to process plasmonic signals, with electrical detection being particularly advantageous for on-chip circuitry. Typically this entails the generation of electron-hole pairs directly by SPPs, or by out-coupled photons, which subsequently gives rise to a photocurrent^18^,^19^,^20^,^21^. VCSELs, as p-n junction diodes, may function as photodetectors when operated in reverse bias, thereby serving dual roles in photonic components. Therefore, VCSELs also have the capability of detecting SPPs, once these modes have been coupled into the diode. While the thin active layer of VCSELs only partially absorbs the incident radiation, the probability of photocurrent generation is increased due to the recycling of light within the cavity^22^,^23^.

**Figure 6:**
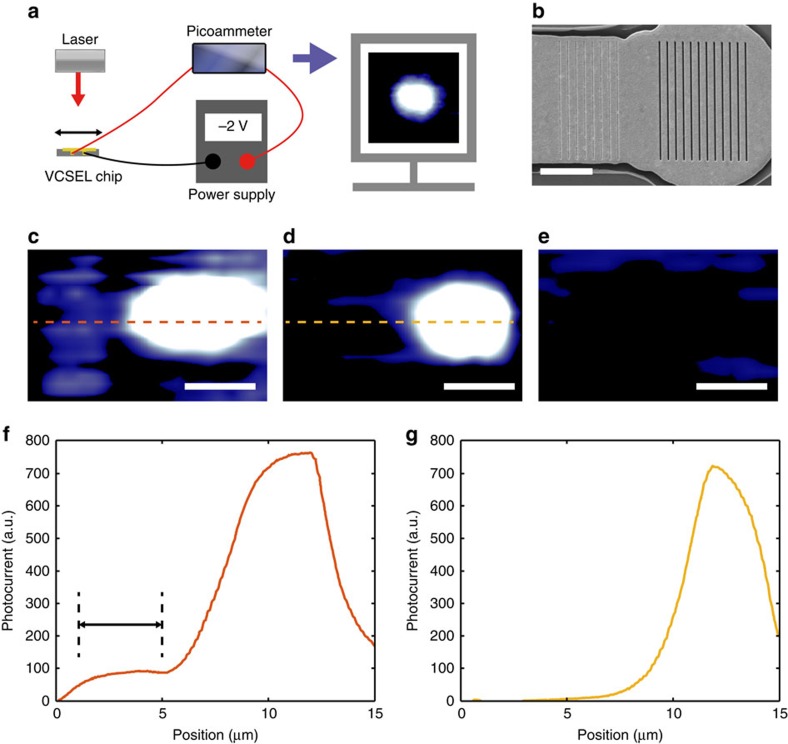
SPP detection by a VCSEL. (**a**) The set-up for detecting spatially resolved photocurrent by raster scanning a diffraction limited laser beam across a reverse-biased VCSEL. (**b**) Scanning electron microscope image of the groove (eight grooves of a 50 nm width with an 800 nm period) and slit (twelve slits of 100 nm width with a 760 nm period) gratings milled into a waveguide and plasmonic VCSEL, respectively. (**c**–**e**) Photocurrent maps recorded with (**c**,**d**) the light polarized perpendicularly to and (**e**) along the grooves and slits for the device with (**c**,**e**) both gratings and (**d**) a slit grating only. (**f**) and (**g**) are the cross-sections of (**c**) and (**d**), respectively, and are marked with dashed lines. The position of the groove grating is indicated in (**f**) with a line. Scale bars, 5 μm in all images. a.u., arbitrary units.

In order to demonstrate SPP detection, two gratings were fabricated on the plasmonic VCSEL: a slit grating, etched over the VCSEL emission area, and a groove grating, positioned on the adjacent waveguide (Fig. 6b). Under external illumination, the groove grating serves to excite an SPP mode that propagates towards the slit grating where it couples into the VCSEL cavity. It is important that the SPP excitation grating on the waveguide be composed of grooves to prevent direct transmission of light into the cavity below the Au layer. In order to provide spatial selectivity, a diffraction-limited laser beam spot from a conventional laser diode emitting at a wavelength of 850 nm was raster scanned across the surface of the waveguide of a plasmonic VCSEL that was operated in reverse bias. The resulting photocurrent from the VCSEL was then mapped as a function of the illumination spot position.

For the light polarized perpendicularly to the grooves, Fig. 6c shows that local illumination of the groove grating, away from the VCSEL emission area, results in photocurrent generation in the VCSEL. This is related to SPP mode excitation and waveguiding towards the slit grating located on top of the emission area, where the plasmonic mode is scattered into the VCSEL cavity, creating a photocurrent. Moreover, when the slit grating is illuminated, direct transmission into the cavity occurred, producing a spot comparable in size to the VCSEL oxide aperture. In contrast, no photocurrent is detected under illumination with light polarized along the grooves, which cannot excite SPP modes (Fig. 6e). Also, the photocurrent map for the device with only a slit grating milled into the VCSEL's plasmonic layer displays only one hotspot, as expected, since the conventional illumination of the waveguide away from the slit grating is unable to efficiently excite SPPs (Fig. 6d). As the signal must couple to the VCSEL cavity in order to be detected, the proposed approach allows for high wavelength selectivity. Additionally, the responsivity of the on-chip plasmonic detector may be controlled by altering the reverse bias.

## Discussion

We have successfully developed a plasmonic VCSEL platform for on-chip nanophotonic circuitry, supporting SPP waveguiding, manipulation, frequency conversion and detection. The design was fabricated by effectively adding a few technologically straightforward steps to a typical VCSEL manufacturing process, making this platform completely compatible with current industrial fabrication methods^24^. Furthermore, the plasmonic VCSELs we have designed may be easily expanded upon—for instance dielectric-loaded^2^,^25^ and slot waveguides^26^ can be conveniently incorporated with gain media to offset SPP attentuation^27^. The modulation of SPP signals is also naturally possible with VCSELs by directly controlling the injection current, offering speeds in the gigahertz range^3^.

With regards to possible applications, plasmonic VCSELs provide an immediate opportunity for realizing on-chip sensing. For instance, two plasmonic VCSELs connected together with an MZI metal waveguide may be envisioned as a simple sensor. In this case, a first diode generates SPPs, with a second diode detecting the plasmonic signal following propagation on the interferometric waveguide. The presence of analyte molecules may thus be inferred from variations in the output VCSEL photocurrent due to changes in the SPP path length. This system effectively constitutes a fully integrated, on-chip circuit, and its fabrication is well within current industrial standards. Other compatible applications include surface-enhanced Raman spectroscopy^28^ and heat-assisted magnetic recording^29^,^30^,^31^,^32^, which may take advantage of the significant local fields sustained by plasmonic excitations, and thus may prove to be major technologies for the proposed VCSEL platform.

## Methods

### VCSEL manufacture

The diode structure was composed of p- and n-doped distributed Bragg reflectors, containing 35.5 and 22 pairs of graded index Al_0.12_Ga_0.88_As–Al_0.9_Ga_0.1_As, respectively. An Al_0.98_Ga_0.02_As layer replaced an Al_0.9_Ga_0.1_As layer in the upper half of the structure, next to the p-type reflectors, for selective oxidation to form an aperture. Sandwiched between the distributed Bragg reflectors was the active region, made up of three 6 nm thick GaAs quantum wells. Mesas of 46 μm in diameter, together with an extended platform that formed the base of the plasmonic waveguide, were lithographically patterned and dry etched. Selective oxidation resulted in 4 μm apertures, thereby determining the area that contributed to light generation, with the platform entirely oxidized such as to prevent any current flow in this region. Once the SiO_2_ layer was opened, the p- and n-type metal contacts (Ti-Pt-Au and Au-Ge-Ni, respectively) were deposited. A lift-off process was then employed to form the Au-Cr-SiO_2_ trilayer. Chromium served as the adhesion layer and also inhibited any substrate SPP propagation that would otherwise occur at an Au–SiO_2_ interface. Moreover, the thickness of the SiO_2_ layer was approximately equal to half of the wavelength in the material, ensuring that an electric field antinode occurred at the boundary with the Cr film.

### Scanning near-field optical microscopy

A custom-built set-up with tapping mode distance regulation was employed using a probe based on a pulled, metallized optical fibre with a nanoaperture. The probe served to scatter near fields with the resulting light also collected by the same probe. The output of this fibre was coupled to a photomultiplier tube, and thus the spatially resolved near-field signal was recorded as the probe was scanned across the sample's surface. This produced a topographic map together with the optical field distribution, allowing correlation between the two. Further details are available in refs 7, 8.

### Numerical modelling

A finite element method (COMSOL Multiphysics 4.3a) was employed to model the stripe waveguides and SPP coupling via the slit-groove grating.

### Data availability

All data supporting this research are available within the article and its Supplementary Information File.

## Additional information

**How to cite this article:** McPolin, C. T. P. *et al*. Integrated plasmonic circuitry on a vertical-cavity surface-emitting semiconductor laser platform. *Nat. Commun.* 7:12409 doi: 10.1038/ncomms12409 (2016).

## Supplementary Material

Supplementary Information Supplementary Figures 1 and 2.
