# Supplementary material for: Integrated plasmonic circuitry on a vertical-cavity surface-emitting semiconductor laser platform
Source: Nat Commun. 2016 Aug 5;7:12409. doi: 10.1038/ncomms12409 (PMC4980450; doi:10.1038/ncomms12409)
Supplement: Supplementary Information — Supplementary Figures 1 and 2. [file ncomms12409-s1.pdf]

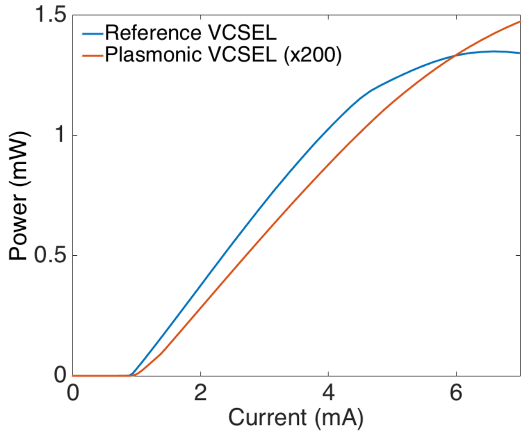

Supplementary Figure 1 I Comparison of the light power – current (LI) curves for reference and plasmonic VCSELs. The reference VCSEL has no metal on the emitting region. The plasmonic VCSEL has a SiO_2/Cr/Au (297/20/200 nm) trilayer and a coupling grating (5 slits of length 8 μm and width 200 nm with a period of 800 nm) over the emitting region. The plasmonic VCSEL intensity has been multiplied by a factor of 200. The LI characteristics show that the intensity of the light emitted in the far-field from the plasmonic VCSEL is reduced by a factor of approximately 200 compared to the reference VCSEL. The threshold currents are the same for both devices.

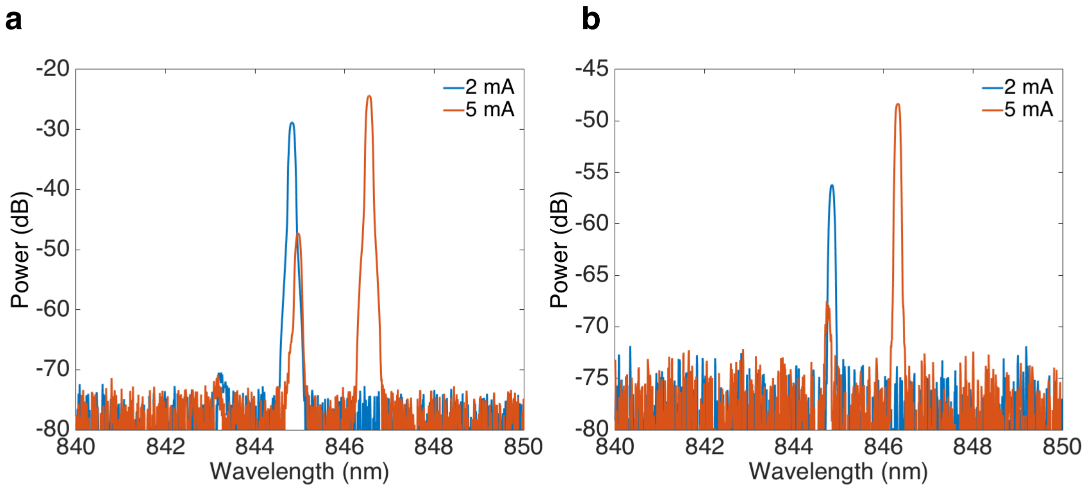

Supplementary Figure 2 I Comparison of the emission spectra of the reference and plasmonic VCSELs. The emission spectra at 2 mA and 5 mA are measured for (**a**) the reference VCSEL and (**b**) the plasmonic VCSEL. The spectra are identical with a higher order transverse mode showing at approximately 5 mA. The shift in wavelength with current is due to Joule heating of the active region.
